# Eccentricity-dependent saccadic reaction time: The roles of foveal magnification and attentional orienting

**DOI:** 10.1016/j.isci.2025.113042

**Published:** 2025-07-01

**Authors:** Yufeng Zhang, Pascal Fries

**Affiliations:** 1 Ernst Strüngmann Institute (ESI) for Neuroscience in Cooperation with Max Planck Society, 60528 Frankfurt, Germany; 2 Donders Institute for Brain, Cognition and Behaviour, Radboud University, 6525 EN Nijmegen, the Netherlands; 3 International Max Planck Research School for Neural Circuits, 60438 Frankfurt, Germany; 4 Max Planck Institute for Biological Cybernetics, 72076 Tübingen, Germany

**Keywords:** Neuroscience

## Abstract

The primate visual brain is characterized by foveal magnification. Here, we show in macaque monkeys that foveal magnification affects the dynamics of saccade initiation. In a visually guided saccade task, saccadic reaction times (SRT) increased with target eccentricity. Notably, we effectively eliminated this increase by scaling the target size according to the foveal magnification factor in the superior colliculus. We then repeated the comparison between non-scaled and scaled targets while changing the task to a delayed, visually guided saccade task. In this task, the saccade was triggered by the foveal fixation offset rather than target onset, such that target onset long before the fixation offset was essentially irrelevant for SRT. In this task, we found that SRT increased with target eccentricity, with a similar rate for both non-scaled and scaled targets, consistent with an attentional scan from the fovea to the target, a recently hypothesized general mechanism of attention.

## Highlights

• For equal-sized targets, saccadic reaction times increase with target eccentricity
• This increase is eliminated by scaling targets according to foveal magnification
• Scaling is most effective for low-contrast targets
• The increase persists in delayed saccades, independent of scaling

## Graphical abstract

**Figure:**
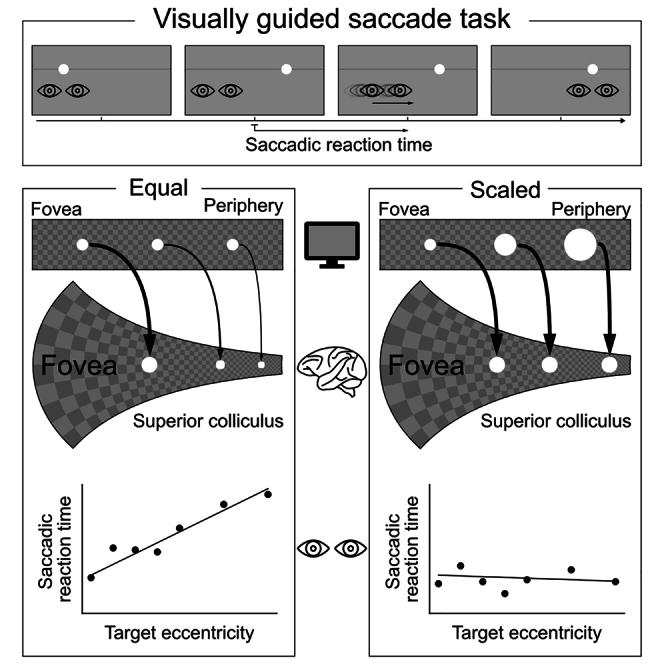

## Introduction

We make several saccades per second, redirecting our gaze to bring objects of interest into our high-resolution fovea. However, the time it takes to initiate a saccade to a novel target, i.e., the saccadic reaction time (SRT) can vary significantly, even when the task is simple and highly repetitive. Part of the variability comes from the size of the impending saccade. The relationship between SRT and the saccade size has a characteristic bowl shape with long SRTs for the shortest (<1 degree of visual angle [dva]) and longest (>10 dva) saccades, and shorter SRTs for medium-sized saccades Kalesnykas and Hallett.^1^^,^^2^^,^^3^^,^^4^^,^^5^ Within these medium-sized saccades, the relationship between SRT and target eccentricity remains unclear and seems task-dependent.^1^^,^^4^^,^^6^^,^^7^^,^^8^^,^^9^ Yet, these medium-sized saccades between 2 and 10 dva constitute the most commonly executed saccades in daily life^10^^,^^11^ and in laboratory settings, where SRTs are widely used as a tool to characterize the cognitive process of interest.^12^^,^^13^^,^^14^

Previous studies describing the relationships between SRT and saccade size often utilized a Step task. In a Step task, the fixation dot steps into the periphery and becomes the saccade target. This design usually comes with a significant confounding factor: saccade targets that are physically identical at various positions vary in visibility with corresponding saccade sizes.^15^ This is partially due to the fact that a visual stimulus of a given physical size undergoes foveal magnification early in the visual processing pathways^16^^,^^17^^,^^18^ and, therefore, has a decreasing drive for increasing eccentricity or saccade size, respectively.^19^ At the single-neuron level, this decreasing drive is correlated with the stimulus occupying a smaller proportion of the receptive field (RF), the size of which increases as the RF moves away from the fovea.^16^^,^^17^^,^^20^ Further, accompanied by foveal magnification and the corresponding change in RF sizes, neurons representing the visual periphery prefer lower spatial frequency components as compared to neurons representing the fovea.^21^ Given that a dot is a broad-spectrum stimulus, its spectral components will be amplified differentially at different eccentricities. In addition, low background lighting commonly employed in previous studies might have resulted in the target onset signal being too strong, such that the SRT showed a floor effect, which might have masked the effect of target eccentricity.^22^ It is, therefore, important to control the strength of the afferent signal at different eccentricities to draw conclusions about the dependence of SRT on target eccentricity *per se*.

Besides physical stimulation, the nature of the impending saccade, as determined by the specific task structure, can also influence the relationship between SRT and target eccentricity. Recently, Hafed and Goffart^4^ reported a clear effect of increasing SRT with target eccentricity in a visually guided saccade task. Despite the confounding factors mentioned earlier, when the same stimulus set was used for a *delayed* visually guided saccade task, the observed increase of SRT with eccentricity became much less prominent. This is interesting because from the perspective of superior colliculus (SC) which plays a major role in saccade initiation, the main difference between these two tasks is that in the visually guided saccade task, the response saccade was driven by localized exogenous input, dominated by the transient onset of the target stimulus; in contrast, in the *delayed* visually guided saccade, the response saccade was driven by voluntary endogenous input, triggered by the fixation dot offset, far away from the target stimulus.^23^ The observation that the SRT in these two tasks exhibited different patterns of eccentricity dependence motivated us to investigate these two types of saccades (endogenous saccades and exogenous saccades) separately.

Given the same physical conditions and task structure, SRTs across trials can still exhibit high variability.^24^^,^^25^ The high variability of SRT requires many trials to reveal a modest shift of the SRT distribution. For example, to reveal a shift of 10 ms in a typical SRT distribution, more than 200 trials per condition are needed (calculated with an ex-Gaussian peaked at 200 ms with a standard deviation of 38 ms; two sided Mann-Whitney U test). Such high trial counts per condition were generally not obtained in previous studies.

**Figure 1:**
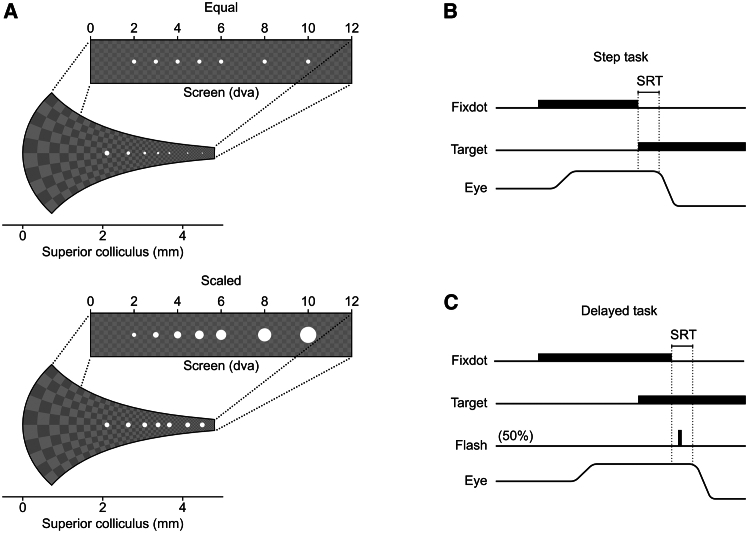
Stimuli and task (A) Representation of target stimuli on the screen and their corresponding afferent input in the SC map, based on the foveal magnification reported in Chen et al*.*^17^ Top: Equal targets at varying eccentricities on the screen exhibit a diminishing size of corresponding afferent input in the SC. Bottom: Scaled targets on the screen have the same corresponding afferent input size in the SC map. Note that the checkerboard background is shown for illustration only and was not presented during the experiment. The target size has also been enlarged to enhance visibility in the figure. (B) In the Step task, the fixation dot stepped into the periphery and became the target stimulus. (C) In the Delayed task, the target presentation preceded the fixation dot removal. The animal was required to wait until the removal of the fixation dot before shifting its gaze. In 50% of trials, a transient (≈30 ms) foveal flash (white-filled circle of 1.0 dva diameter) was presented at ≈100 ms after fixation dot removal. The flash had no task relevance and served to capture attention back to the fovea, while the response saccade was planned but not yet executed.

The current study aims to address the aforementioned issues in macaque monkeys because they offer a rich history of studies of oculomotor control and attention, and also the potential to use the current results to motivate future circuit investigations. We selected two sets of stimuli as saccade targets located between 2 and 10 dva (Figure 1A). One set is Equal, having the same physical size at different eccentricities. The second set is Scaled, with the physical sizes scaled based on the foveal magnification in the SC, aiming to equalize the targets’ corresponding afferent input strength to the SC across eccentricities.^17^ To avoid a flooring effect due to high input strength, in all tasks, we presented the target on a gray background. We used the same stimuli sets in both, a Step (visually guided saccade) task where the response saccade is exogenously driven by the target onset, and a Delayed (delayed visually guided saccade) task where the response saccade is endogenously triggered by the fixation dot offset (Figures 1B and 1C). Because the scaling is supposed to mainly affect the target onset transient, we expect to see the scaling to reduce SRT significantly in the Step task but to affect SRT minimally in the Delayed task. Additionally, in the Delayed task, we incorporated an extra condition including a transient attention-capturing distractor flash to investigate whether the eccentricity-dependent SRT changes for the endogenously driven saccades are consistent with an attentional scan from the fovea. Last but not least, for each monkey and each condition, we collected more than 200 trials per condition to gain the necessary statistical power.

## Results

### Foveal magnification explains eccentricity-dependent saccadic reaction times increase for exogenously driven saccades

We start with exogenously driven saccades in the Step task (Figure 1B). The hypothesis is that, for exogenously driven saccades, the reported SRT increase with eccentricity is due to foveal magnification.^4^ Specifically, foveal magnification, i.e., the increasing neural tissue devoted to stimuli closer to the fovea, is accompanied by decreasing RF sizes of neurons with decreasing eccentricity. This inverse relationship between RF sizes and magnification factor equalizes the size of the active neuronal population for the same targets located at different eccentricities.^16^^,^^17^ Increasing RF size with increasing eccentricity, however, decreases the visibility of the stimulus because it occupies a smaller portion of the (excitatory) RF. This hypothesis also aligns with the recently reported observation that SC neurons at higher eccentricities prefer lower spatial frequencies (corresponding to a larger dot).^21^

To test this hypothesis, we designed two stimulus sets as saccade targets. The first set, Equal, was similar to what has been used in previous studies^4^^,^^6^ (Figure 1A, top). In this set, saccade targets at all positions were white filled circles of diameter 0.1 dva. The second set, Scaled, consisted of filled white circles whose sizes were scaled according to the reported magnification factor in the SC, such that they provided the same afferent input measured in the SC map (Figure 1A, bottom).^17^ Both sets consisted of stimuli positioned at 2, 3, 4, 5, 6, 8, and 10 dva to the right of the fixation point along the horizontal median. To facilitate comparison, the target stimulus at 2 dva was of the same size in both sets.

Figure 1B illustrates the task structure of the Step task. In this task, each trial started with the monkey maintaining fixation on a fixation dot (0.1 dva filled white circle) for 800 to 1500 ms. Subsequently, the fixation dot was turned off. At the same video frame, a single saccade target randomly chosen from the combined Equal and Scaled stimuli set was presented. The monkey needed to make a saccade within 500 ms toward the target and hold its gaze on the target for another 800 to 1500 ms. The saccade landing on the target was determined by the gaze entering in a visual target window (r ≈ 1.0 dva) centered on the saccade target. If the animal completed the trial correctly, a juice reward was provided. Only correct trials were included in the analysis.

**Figure 2:**
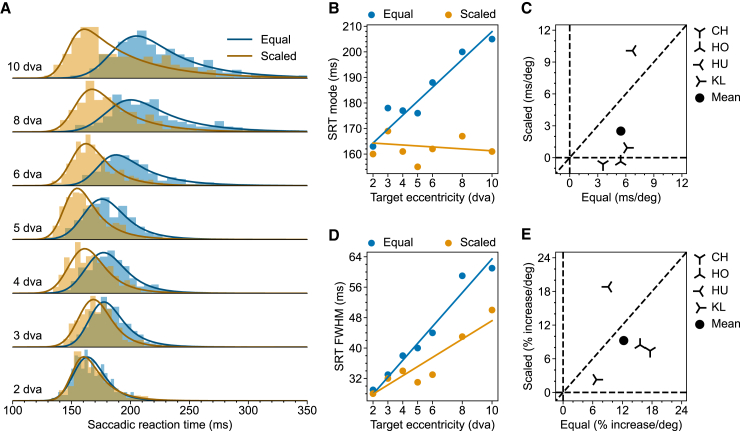
Eccentricity-dependent SRT in the Step task (A) SRT distributions from monkey HO for Equal (Blue) and Scaled (Orange) targets. Solid lines represent the fitted ex-Gaussian distributions. Saccade landing positions are presented in the supplementary material (Figure S1). (B) Multiple linear regression of SRT mode on target eccentricity and scaling for monkey HO. (C) Scatterplot of SRT mode-eccentricity slopes for Scaled vs. Equal targets in all tested monkeys. For each monkey, the slopes are derived from the regression coefficient, as demonstrated in (B). The filled circle shows the mean slopes. (D) Same as (B) but for FWHM. (E) Same as (C) but for FWHM. The raw slopes were converted to percent increase per degree of visual angle. Results for individual monkeys other than HO are provided in the supplemental information (Figures S2–S4).

The resulting SRT distributions in each condition from one example monkey (HO) are plotted in Figure 2A. The raw data show that for this monkey: 1) Both location and shape of the SRT distribution varied across conditions. 2) Similar to what has been reported before,^4^ the peak of the SRT distributions for Equal targets (blue) gradually increased with increasing target eccentricity. 3) This shift was significantly reduced for Scaled targets (orange). 4) SRTs also became more variable as they became longer. Noteworthy is that the saccade landing position became more variable across trials for more eccentric target, however, there is no systematic difference between saccades to the Equal and Scaled target (Figure S1). Next, we quantified these observations for the SRT distributions.

To quantify these observations, we first fitted an exponentially modified Gaussian (ex-Gaussian, solid lines in Figure 2A) for each SRT distribution. The ex-Gaussian is the most commonly used parametric function to describe a reaction time distribution.^26^^,^^27^ We used the mode of each resulting ex-Gaussian fit to represent the typical SRT, as it denotes the most likely occurring value in the distribution. Additionally, we used the full width at half maximum (FWHM) to represent the SRT variability. Having these values, we first examined how the typical SRT varied with saccade size, separately for Equal and Scaled targets. To this end, we fitted a simple linear regression model ($Mode\;∼\;{\beta_{0}+\beta }_{1}*\left(targetEccentricity-2\right)$. For monkey HO, SRT mode increased with target eccentricity for the Equal targets but not for the Scaled targets (${Equal,CI}_{\beta_{1},95\%}=\left[4.33,5.73\right],p<.001;\;{Scaled,CI}_{\beta_{1},95\%}=\left[-0.55,0.71\right],p=.82$). Next, to confirm that scaling reduced the slope of SRT as a function of target eccentricity, we fitted a multiple linear regression model ($Mode∼\;\beta_{0}+\beta_{1}*\left(targetEccentricity-2\right)+\beta_{2}*\left(targetEccentricity-2\right)*isScaled$) on the combined data from Equal and Scaled targets (Figure 2B). Scaling indeed reduced the slope (${Combined,CI}_{\beta_{2},95\%}=\left[-6.45,-5.25\right],p<.001$). We repeated the same procedure for the other three monkeys (Figures S2–S4). The resulting slopes for each monkey in both conditions are plotted in Figure 2C. Notice that data from three out of four monkeys (CH, HO, and KL) lie very close to the horizontal zero line meaning that, for these monkeys scaling the target stimulus according to the SC foveal magnification factor effectively eliminated the eccentricity-dependent SRT increase. Having only four monkeys, we are essentially limited to the fixed effect analysis and draw inferences on these four monkeys instead of on the population.^28^ Fixed effect analysis shows that for these four monkeys, scaling significantly reduced the average slope (${\;CI}_{\bar{\beta_{2}},95\%}=\left[-3.42,-2.59\right],p<.001$). We repeated the same analysis for the variability of SRT (ex-Gaussian FWHM). Similarly, for monkey HO, FWHM increased with target eccentricity for Equal targets ($Equal{\;CI}_{\beta_{1},95\%}=\left[3.43,5.11\right],p<.001$). Scaling also reduced the speed of increase for FWHM but this reduction was not as effective as for the mode (Figure 2D, ${\;{\;Scaled,CI}_{\beta_{1},95\%}=\left[1.84,3.24\right],p<.001;\;Combined,CI}_{\beta_{2},95\%}=\left[-2.80,-1.37\right],p<.001$). The results for the other three monkeys are presented in the supplemental information (Figures S2–S4). Fixed effect analysis using averaged percent increase per degree on FWHM agreed with the pattern observed in monkey HO (Figure 2E, ${\;CI}_{\bar{\beta_{2}},95\%}=\left[-4.41,-1.95\right]\text{,}$ p=.001).

### Scaling reduces saccadic reaction times increase most effectively for low-contrast targets

The results in the previous section suggest that the observed SRT increase with increasing target eccentricity could be explained by foveal magnification. This is presumably because, accompanied by foveal magnification, RF size increases as the RF moves away from the fovea.^16^^,^^17^ Consequently, a physically identical stimulus closer to the fovea would drive the corresponding neuron stronger in the SC because it occupies a larger proportion of the RF. This stronger stimulus drive would then result in a faster saccade initiation.^21^ Also, a stimulus with higher contrast evokes a stronger and faster response throughout the visual hierarchy, leading to a faster reaction time.^29^^,^^30^^,^^31^^,^^32^^,^^33^^,^^34^ Furthermore, these two bottom-up factors, stimulus size and stimulus contrast, are often found to interact in such a way that, at higher contrast, increasing stimulus size is less effective in driving the neurons stronger. In surround modulation, a widely observed property of visual neurons, this interaction is reflected in the fact that the peak of the size tuning curve shifts toward larger stimuli at lower contrast.^35^ In the SC, besides surround suppression, summation within the RF center seems to occur effectively only for targets of low contrast.^36^^,^^37^ We therefore examined the interaction between stimulus size and contrast in the eccentricity-dependent SRT increase.

To this end, we slightly modified the Step task that we had used so far. Firstly, we reduced the screen background luminance to allow for a wider range of target contrast manipulation. Secondly, at each target location and for each target size, we selected three target luminance levels such that the resulting contrasts were approximately evenly spaced on a logarithmic scale, with the medium contrast targets having the same Weber contrast as the saccade targets used in the original Step task (Weber contrasts of 1.4, 3.1 and 6.9 for low, medium, and high contrast targets respectively). Lastly, we limited target locations to 2, 4, and 6 dva to ensure an adequate number of trials for each condition. We collected data from one monkey (HO) for this modified Step task.

**Figure 3:**
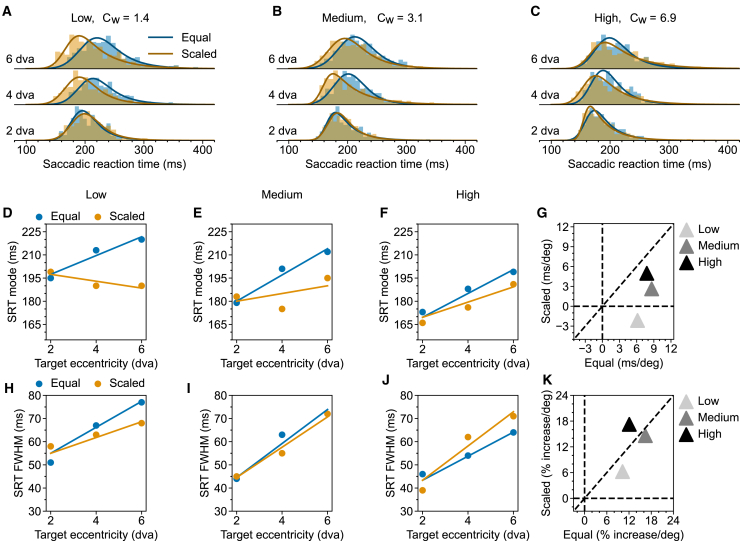
Eccentricity-dependent SRT for targets of different contrast in Monkey HO (A–C) SRT distributions for low-, medium-, and high-contrast targets are shown separately for Equal (blue) and Scaled (orange) targets. Solid lines represent the fitted ex-Gaussian distributions. Weber contrasts, C_W_, for low-, medium-, and high-contrast targets are 1.4, 3.1, and 6.9, as also listed on top of each column. (D–F) Multiple linear regression of SRT mode on target eccentricity and scaling for the low-, medium-, and high-contrast, respectively. (G) Scatterplot of SRT mode-eccentricity slopes for Scaled vs. Equal targets at all contrast levels. (H–K) Same as (D–G), but for FWHM.

We plotted the resulting SRT distributions in Figures 3A–3C. Compared to the original Step task, the modified version led to a general increase in SRTs. For medium-contrast targets, SRTs increased by approximately 20 ms at each location (Figures 3B and 3E vs; Figures 2A and 2B). This general increase is probably due to the reduced screen background lighting, which may have lowered the animal’s alertness. Despite this general increase, SRT for Equal targets tended to be longer at more peripheral locations at each tested contrast level. Scaling reduced this eccentricity-dependent SRT increase. However, the effectiveness of scaling appeared to decrease with increasing contrast. As before, we used linear regression to quantify these observations (Figures 3D–3K).

To evaluate how SRT mode depends on target eccentricity, we first fitted a simple linear regression model ($Mode\;∼\;{\beta_{0}+\beta }_{1}*\left(targetEccentricity-2\right)$) at each contrast level, separately for the scaled and equal targets. For the Equal targets, the SRT mode increased with target eccentricity at each contrast level. For the Scaled targets, the SRT mode decreased slightly with eccentricity at low contrast and increased with eccentricity at higher contrast. At each contrast level, the slopes are lower for the Scaled compared to the Equal targets. This difference in SRT-Eccentricity slopes between Scaled and Equal targets indexes the effectiveness of scaling. To quantify the effectiveness of scaling reducing the slope, we then fitted a multiple linear regression model ($Mode\;∼\;{\beta_{0}+\beta }_{1}*\left(targetEccentricity-2\right)+\beta_{2}*\left(targetEccentricity-2\right)*IsScaled$), combining data from Equal and Scaled targets (Figures 3D–3F) at each contrast level. At low and medium contrast, scaling reduced the slope significantly, but the reduction of slope due to scaling was marginal for high-contrast targets ($Low\;contrast,{\;CI}_{\beta_{2},95\%}=\left[-10.30,-6.00\right],p<.001;\;Medium\;contrast,{\;CI}_{\beta_{2},95\%}=\left[-8.40,-3.80\right],p<.001;{High\;contrast,CI}_{\beta_{2},95\%}=\left[-5.80,1.20\right],p=.063$). The above results from the multiple linear regression analysis suggest that the effectiveness of scaling decreased at higher target contrast. This trend is illustrated graphically in Figure 3G: with increasing contrast, data points moved gradually toward the equal-slope diagonal. As before, we applied the same analysis to FWHM (Figures 3H–3K). FWHM increased with more peripheral targets for both Equal and Scaled targets. Stimulus scaling had a smaller effect on FWHM. It reduced the slope only marginally for the low-contrast targets ($Low\;contrast,{\;CI}_{\beta_{2},95\%}=\left[-4.30,0.50\right],p=.099;\;Medium\;contrast,{\;CI}_{\beta_{2},95\%}=\left[-3.10,2.30\right],p=.537;{High\;contrast,CI}_{\beta_{2},95\%}=\left[-.60,5.90\right],p=.171$).

### Eccentricity-dependent saccadic reaction times increase for endogenously driven saccades is consistent with an attentional scan from the fovea

So far, we focused on saccades in the Step task driven by the appearance of a peripheral saccade target, which occurs simultaneously with the disappearance of the fixation point; we refer to these as exogenously driven saccades. In this section, we switch to the Delayed task, where the response saccades were driven by the disappearance of the fixation point after the peripheral saccade target had already been present for 800–1500 ms, and we refer to these as endogenously driven saccades (Figure 1C). The same two stimulus sets, Equal and Scaled, with filled white circles, were used as target stimuli. Like the Step task, each trial in the Delayed task started with the monkey holding fixation on a fixation dot for 800 to 1000 ms. The target stimulus was then presented for 800 to 1500 ms. Importantly, during this period, the monkey was required to keep fixation on the fixation dot and restrain from making a saccade. Next, the fixation dot was turned off, which signaled the monkey to make a response saccade to the target stimulus. The monkey needed to shift its gaze to the target stimulus within 500 ms and hold its gaze on the target for another 800 to 1500 ms to complete the trial and receive a reward. Only correct trials were included in our analysis. Additionally, in 50% of the trials, we included a short ≈30 ms flash at the fovea (1.0 dva diameter filled white circle) starting ≈100 ms after the fixation dot offset. The flash had no task relevance and was intended to capture attention back to the fovea when the response saccade was planned but, in most trials, not yet executed.^38^ We collected data from three monkeys (KL, CH, and HO) for this task. Monkey HO was excluded from training and data collection for the Delayed task because it lacked the effects observed in the Step task in the other monkeys, which we intended to investigate further in the Delayed task.

**Figure 4:**
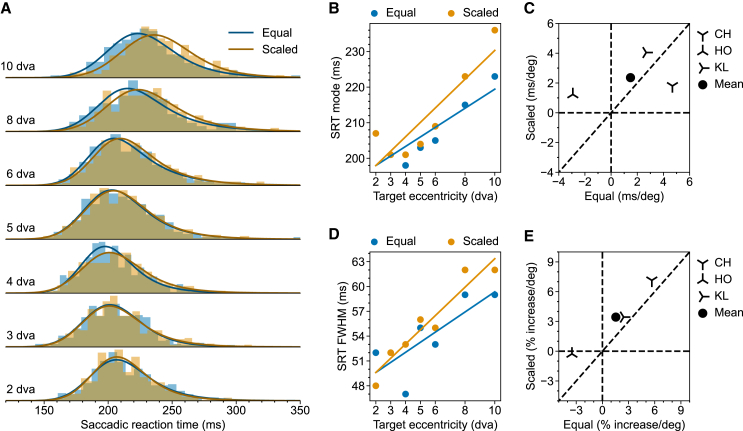
Eccentricity-dependent SRT in the Delayed task without foveal flash (A) SRT distributions from monkey KL for Equal (Blue) and Scaled (Orange) targets. (B) Multiple linear regression of SRT mode on target eccentricity and scaling for monkey KL. (C) Scatterplot of SRT mode-eccentricity slopes for Scaled vs. Equal targets in all tested monkeys. For each monkey, the slopes are derived from the regression coefficient, as demonstrated in (B). The filled circle shows the mean slopes. (D) Same as (B) but for FWHM. (E) Same as (C) but for FWHM. The raw slopes were converted to percent increase per degree of visual angle. Results for individual monkeys other than KL are provided in the supplemental information (Figures S5 and S6).

We first present results from trials where no flash was presented. Figure 4A shows the SRT distributions from monkey KL. These SRT distributions are clearly different from the SRT distributions obtained in the Step task, wherein saccades are more reflexive. First of all, SRT in the Delayed task is generally longer. Second, SRT in the Delayed task is generally more variable. Last but not least, the effect of scaling was much smaller, with the SRT distribution for Scaled targets and Equal targets almost entirely overlapping for targets up to 8 dva. There is actually a small effect of scaling on increasing SRT. All three observations are consistent with the fact that the sustained stimulus drive after the initial transient response is small and insensitive to the target size. It is worth noting that a larger stimulus may invoke lower sustained activity in the SC neurons even though its evoked transient response is larger, which agrees with the observed effect of scaling increasing SRT in the Delayed task.^36^

As before, we used linear regression to quantify how the mode and FWHM of SRT distributions change with target eccentricity and stimulus scaling. Simple linear regression shows that with monkey KL, SRT mode increased with target eccentricity for both Equal and Scaled targets (${Equal,CI}_{\beta_{1}}=\left[2.00,3.22\right],p<.001;\;{Scaled,CI}_{\beta_{1}}=\left[3.63,4.75\right],p<.001\text{;}$). Interestingly, scaling slightly increased the slope, showing the opposite effect as in the Step task (Figure 4B, ${\;CI}_{\beta_{2},95\%}=\left[.85,1.91\right],p<.001$). The other two animals showed some variability in these effects. Specifically, for monkey CH, SRT increased with target eccentricity for both Equal and Scaled targets; scaling decreased the slope (Figure S5), an effect largely driven by the Scaled target at 10 dva. For monkey HO, SRT decreased with target eccentricity for Equal targets; scaling increased the slope. The SRT decrease for Equal targets is driven by the initial decrease of SRT for targets at eccentricities below 4 dva (Figure S6). Despite this variability across animals, fixed-effect analysis pooling the three monkeys tested in this task showed similar results as in the example monkey KL (Figure 4C, ${\;{Equal,CI}_{\bar{\beta_{1}},95\%}=\left[1.50,2.66\right],p<.001;\;{Scaled,CI}_{\bar{\beta_{1}},95\%}=\left[1.08,2.35\right],p<.001;\;CI}_{\bar{\beta_{2}},95\%}=\left[0.32,1.41\right],p<.001$). We repeated the same analysis for FWHM and the results is qualitatively the same as for the mode (Figure 4D, ${Equal,CI}_{\beta_{1}}=\left[.51,2.20\right],p=.009;\;{Scaled,CI}_{\beta_{1}\;}=\left[.90,2.52\right],p<.001$Figure 4E, ${\;{Equal,CI}_{\bar{\beta_{1}}}=\left[1.06,2.84\right],p<.001;{Scaled,CI}_{\bar{\beta_{1}}\;}=\left[1.99,3.92\right],p<.001;\;CI}_{\bar{\beta_{2}}}=\left[1.03,2.72\right],p<.001$).

The observed increase of SRT with target eccentricity in the Delayed task is intriguing. Since the delay between target onset and fixation dot offset was at least 800 ms, the monkey had enough time to prepare for the impending saccade. The increase is therefore probably not related to motor planning before the “Go” signal. Rather, it is likely related to processes that happened after the “Go” signal. Additionally, because the effect is eccentricity dependent, it is likely caused by processes that are spatially relevant. One potential candidate is pre-saccadic attention shift. Note that 1) because the “Go” signal in the Delayed task is the fixation dot offset, to make a timely response, the animal needed to pay attention to the fovea, even though there were two stimuli (fixation dot and peripheral target) presented at the same time, 2) before actual saccade execution, attention is obligatorily shifted to the location of the saccade target.^39^ Thus, the observed increase of SRT with target eccentricity can be explained if the shift of attention from the fovea to the saccade target takes longer for more eccentric targets.^40^

To provide further evidence that the eccentricity-dependent SRT increase in the Delayed task is related to the shift of attention from the fovea to the saccade target after the “GO” signal, in 50% of the trials, we presented a flash at the fovea, at ≈100 ms after the fixation-point offset (Figure 1C). When presented, this flash would drag attention back to the fovea and force a restart of the relevant processes. If the eccentricity-dependent SRT increase is indeed due to the shift of attention from the fovea to the saccade target, the additional flash stimulus would maintain this increase, albeit with a constant delay in SRT at all target locations.

**Figure 5:**
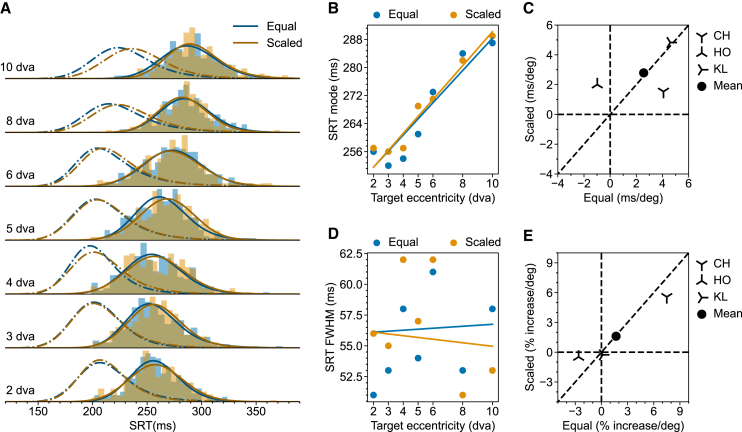
Eccentricity-dependent SRT in the Delayed task with foveal flash (A) SRT distributions from monkey KL for Equal (Blue) and Scaled (Orange) targets. Dashed lines represent the ex-Gaussian fits for SRT distributions in corresponding conditions without the foveal flash (as in Figure 4). (B) Multiple linear regression of SRT mode on target eccentricity and scaling for monkey KL. (C) Scatterplot of SRT mode-eccentricity slopes for Scaled vs. Equal targets in all tested monkeys. For each monkey, the slopes are derived from the regression coefficient, as demonstrated in (B). The filled circle shows the mean slopes. (D) Same as (B) but for FWHM. (E) Same as (C) but for FWHM. The raw slopes were converted to percent increase per degree of visual angle. Results for individual monkeys other than KL are presented in the supplemental information (Figures S7 and S8).

Figure 5A shows the SRT distributions in trials with foveal flash from the same monkey (KL) presented in Figure 4. To facilitate comparison, we plotted the ex-Gaussian fits obtained in corresponding conditions without flashes as dashed lines. As expected, the addition of the flash prolonged the SRT. Importantly, the eccentricity-related SRT increase persisted with the addition of the flash.

We quantified these observations with linear regression. For monkey KL, SRT mode increased with target eccentricity for both Equal and Scaled targets (${Equal,CI}_{\beta_{1}}=\left[4.34,5.49\right],p<.001;\;{Scaled,CI}_{\beta_{1}}=\left[3.98,5.10\right],p<.001$). Notably, after introducing the resetting foveal flash, which presumably further eliminated the differential activity build-up during the delay period, there is now no scaling-dependent change in slope (Figure 5B, ${\;CI}_{\beta_{2}}=\left[-.22,.71\right],p=.356$). Intriguingly, the foveal flash also eliminated the eccentricity-dependent FWHM increase (Figure 5D, ${\;{Equal,CI}_{\beta_{1}}=\left[-.32,1.45\right],p=.223;\;{Scaled,CI}_{\beta_{1}\;}=\left[-1.40,.26\right],p=.163;\;CI}_{\beta_{2}}=\left[-.91,.60\right],p=.586$). The results for the other monkeys are presented in the supplemental information (Figures S7 and S8). Fixed effect analysis combining the three monkeys shows a similar pattern as described above for Monkey KL (Figure 5C, the mode: ${\;{Equal,CI}_{\bar{\beta_{1}}}=\left[\;2.63,3.50\right],p<.001;{\;Scaled,CI}_{\bar{\beta_{1}}\;}=\left[1.88,2.87\right],p=.016;\;CI}_{\bar{\beta_{2}},95\%}=\left[-.1.8,.58\right],p=.274\text{;}$Figure 5E, FWHM ${{Equal,CI}_{\bar{\beta_{1}}}=\left[1.14,3.32\right],p<.001;{Scaled,CI}_{\bar{\beta_{1}}}=\left[.16,2.15\right],p=.016;\;CI}_{\bar{\beta_{2}},95\%}=\left[-.90,.81\right],p=.876$).

## Discussion

Saccadic eye movements play a fundamental role in organizing our spatial and temporal visual input, to the extent that SRT has become a crucial psychophysical measurement enabling us to characterize the underlying cognitive processes that would otherwise remain unseen. However, despite the importance of this measurement, some fundamental questions regarding the determinants of SRT remain, even in relatively simple situations. In this study, we examined the dependence of SRT on target eccentricity in macaque monkeys.

When designing the experiment, we based our hypotheses on previous insights into the role of the SC in generating the response saccades: Once the SC decision unit passes the response threshold, there is a relatively fixed delay in the downstream motor nuclei before the actual eye movement occurs.^41^^,^^42^ Consequently, any observed dependence of SRT on target eccentricity can be attributed to the spatiotemporal activation pattern of the SC and reflects the properties of the neural processes associated with that particular task. It is these properties that have clear behavioral correlates, namely SRT, the primary interest of the current study.

### Saccadic reaction times in the step task

**Figure 6:**
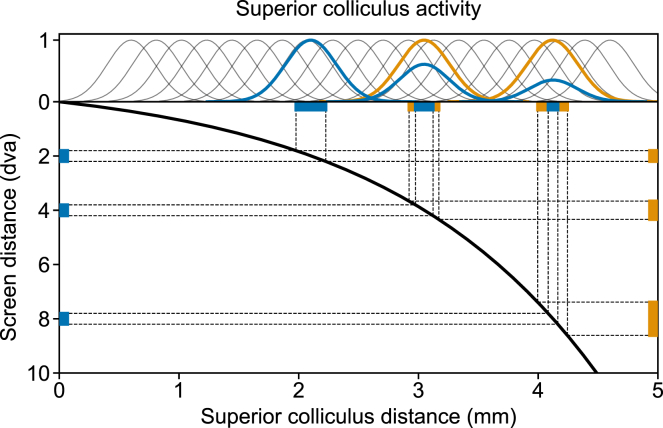
Proposed mechanism of how stimulus scaling reduces SRT in the Step task The foveal magnification is illustrated by the thick black curve mapping points on the screen (Y-axis) to points on the SC (X-axis). Blue (orange) bars on the left (right) represent the Equal (Scaled) targets at 2, 4, and 8 dva, respectively. For illustration purposes, the size of the targets has been enlarged to enhance visibility. The corresponding image of these stimuli in the SC is represented by horizontal blue (orange) bars at the top. These stimulus images represent the foveally magnified afferent input to the SC. The thin gray Gaussian curves on the top illustrate example visual connectivity functions of SC neurons integrating the afferent inputs. Notice that homogeneous connectivity functions, as we assumed here, correspond to RFs of increasing size with higher eccentricity when projected to the visual space. The activity level of a given SC neuron is modeled as the dot product between its Gaussian connectivity function and the afferent input. The activity profile across the SC-neuron population is calculated as the convolution between the connectivity function and the afferent signal. The thick colored curves on the top illustrate the resulting activity profiles evoked by each stimulus. Note that equally sized stimuli (blue) activate the SC more weakly in the periphery, whereas scaled stimuli (orange) activate the SC equally strongly at all positions. Note also that the sizes of the activated neuronal populations are approximately equal because the stimuli are small compared to the SC neurons’ RFs.

In the Step task, afferent input to the SC was presumably dominated by the transient onset of the target stimulus. We first confirmed the previous finding that, for medium-sized saccades, SRT increased with target eccentricity. Notably, this SRT increase was largely explained by the foveal magnification factor in the SC (Figure 2). The observed effect of foveal magnification on SRT can be explained by the increase of the accompanied RF size from the foveal to the peripheral representation in the SC. This increasing RF size compensates for the foveal magnification, thus equalizing the size of the active population for a point stimulus presented at different eccentricities, achieving a constant point image in the SC.^16^^,^^43^ However, larger RFs in the periphery also mean that an equal-sized stimulus occupies a smaller proportion of the corresponding SC neurons’ RFs. The smaller occupied RF proportions likely drive the active population less strongly, leading to longer SRT.^36^ When we scaled the stimulus size according to the SC magnification factor, the above-mentioned mechanism would predict an equal SRT at all eccentricities, which is what we found for the Scaled targets. This mechanism is illustrated in Figure 6. A similar effect of foveal magnification neutralizing the eccentricity effect has been reported for a covert visual search task.^44^

Furthermore, data from monkey HO in the modified Step task suggests that the stimulus scaling becomes less effective with targets of higher contrast (Figure 3). High-contrast targets are more visible, which leads to the faster reaction times. Importantly, the suggested mechanism based on foveal magnification implies that the *relative* visibility of the Equal targets at different locations should remain invariant across contrast levels. Therefore, in the modified Step task, one would expect the SRT to increase with target eccentricity at each tested contrast level. This was indeed what we observed. However, one would also expect that scaling the target size should equalize the target visibility at each contrast level, and hence, flatten the SRT-eccentricity curve equally well. This was not the case. We found that scaling was less effective at higher contrasts. A parsimonious explanation, without modifying the rationale of the proposed mechanism, is that under high contrast, increasing the target size no longer increases the activation level of the underlying neuronal population. In other words, to explain our data, there needs to be less summation over the RF for the high-contrast targets. Indeed, Goldberg and Wurtz^37^ found little summation in the macaque SC when using light spots of varying sizes. Notably, the luminance of their stimuli was 1.5 log units above the background, considerably higher than our high-contrast targets, which were 0.84 log units above the background. For the arguments’ completeness, it is also necessary to show that summation does exist in the SC with low-contrast stimuli. More recently, using grating stimuli, Chen and Hafed^36^ showed that response summation in the SC neurons is contrast dependent. They showed that for a given SC neuron, a large stimulus filling the RF evokes a larger response only if the contrast is low. When the contrast is high (≥20% Michelson contrast), the response magnitude is similar for both small (0.5–1 dva in size) and large stimuli. One may note that our low-contrast targets had a Michelson contrast of 41.7%, falling within the high-contrast range reported by Chen and Hafed.^36^ However, in addition to the differences in stimulus type (light spots vs. gratings), our scaled targets were smaller than the smallest gratings used in Chen and Hafed.^36^ Consequently, a higher contrast is likely needed to provide an equivalent drive.

Our analysis has been focused on comparing SRT across different eccentricities to test whether a particular kind of scaling, i.e., scaling according to the foveal magnification in the SC, affected this SRT-eccentricity relationship. Conversely, one could also compare SRT at fixed target eccentricity across different scaling factors. If our hypothesis is true, that the relative afferent input strength determines the SRT-eccentricity relationship in the Step task, and that the relative size of the saccade target to the corresponding neurons’ RF can approximate this afferent input strength, one would expect that increasing the size of the saccade target at a fixed eccentricity shortens SRT. Indeed, this expected effect of varying saccade target size on SRT was reported for express saccades (latency <100 ms) in a Gap task, toward targets that are small relative to the SC neuron’s RF size.^45^ Different processes appear to be involved in saccading to large targets similar to or larger than the RF size,^46^^,^^47^ which may engage the suppressive surround of SC neurons.^20^^,^^37^

Scaling the target has an incidental consequence: a scaled target is of a different size than the fixation dot and all the non-scaled saccade targets, making it a relatively novel stimulus. Stimulus novelty can decrease reaction time.^48^ However, for the difference in stimulus novelty to cause the observed effect of target scaling, i.e., flattening the SRT-eccentricity function, an additional hypothesis would be required to assign quantitatively appropriate levels of novelty to target stimuli of different sizes. Nevertheless, future research would benefit from using neutral fixation points of different types than the saccade targets to alleviate this concern.^49^

### Saccadic reaction times in the delayed task

In the Delayed task, at the time when the monkey was about to make the response saccade, the target had already been on the screen for at least 800 ms, long beyond the target-onset-related visual burst in the SC neurons, which diminishes around 100 ms after the stimulus onset.^37^^,^^50^ As a result, the afferent input relevant to the response saccade was no longer dominated by the transient onset of the saccade target. In agreement with this prediction, in the Delayed task, the dependence of SRT on eccentricity was minimally affected by scaling (Figures 4A and 5A). Yet, the simple dependence of SRT on eccentricity was still present, leading to the question: What could be the source of this SRT increase? One potential candidate is a pre-saccadic attention shift after the “Go” signal.

Just before the “Go” signal, attention is likely on the fixation point. This is because the saccade target, though initially salient, has been uninformative for ≥800 ms, while the unpredictable fixation offset, serving as the “Go” signal, requires close monitoring. Additionally, it is established that attention obligatorily moves to the saccade target just before saccade execution, even when cued elsewhere.^39^^,^^51^ Therefore, we expect attention to shift from the fixation point to the saccade target after the “Go” signal. If this shift takes longer for more eccentric targets, it can explain the observed SRT increase. Trials with the foveal flash provided further support for this hypothesis. Because abrupt stimulus onset involuntarily captures attention, and this attentional capture is spatially confined, the foveal flash in these trials is expected to explicitly drag attention back to the fovea and restart the relevant shift process.^52^^,^^53^ If the shift process underlies the observed SRT increase, the flash should cause a constant delay at all locations while preserving the SRT increase for more eccentric targets. This is what we observed (Figure 5). A constant delay may also result from saccadic inhibition (SI), a likely low-level transitory oculomotor effect triggered by the foveal flash and time-locked to its onset.^54^ SI typically reduces the saccade rate between 60 and 130 ms after a transient visual event.^55^ The chosen flash onset latency (≈100 ms) caused the baseline SRT distribution to overlap with this window. Future studies could address this issue by using a shorter flash onset latency to alleviate the influence of SI.^54^ In addition, including conditions with the attention-capturing flash placed ipsilaterally may be helpful. An ipsilateral flash likely induces SI with a similar time course as the foveal flash^56^^,^^57^ but systematically changes the sequence of distances that the captured attention needs to shift to arrive at the saccade target.

Whether the pre-saccadic shift of attention in the Delayed task is analog or discrete is not essential for the above argument. Guided by the spotlight metaphor, whether the shift of *covert* attention from position A to position B is analog or discrete has been heatedly discussed in the 1970s and 1980s (see Chapter 3 in Wright and Ward^58^ for a recent review). Central to this debate is whether the intermediate locations between position A and B receive attentional benefit before the benefit emerges at the destination position B. An analog shift of the attentional spotlight would predict this is the case; by contrast, a discrete shift of attentional spotlight would predict this is not the case. Our tasks did not test attentional benefits at these intermediate locations. Consequently, our data cannot speak to the nature of the shift being either analog or discrete. Notably, the observed SRT increase in the Delayed task is compatible with both a discrete shift and an analog shift. Specifically, if the shift is discrete as in Posner’s “Disengage-Shift-Engage” model,^59^ the observed SRT increase can be explained if the shift’s physiological implementation, which Posner has hypothesized to be mediated by the SC,^60^ takes longer if it is shifting toward more eccentric targets. On the other hand, if the shift is analog, the observed SRT increase can also be explained if the analog shift has a constant speed profile independent of the target location. Interestingly, a constant-speed attention shift can be mediated by a spatially smooth wave in the neuronal map, which corresponds to a spatially smooth shift of attention through visual space. If the observed SRT increase is indeed related to pre-saccadic attention shift mediated by a wave in the neuronal map, the regression slopes will inform us about how fast attention was moving from the fovea to the peripheral targets: ≈2.6 ms/dva, as the average from three monkeys (Figure 5). This speed is likely beyond the resolution of previous contested studies which intended to derive the speed of attention shift by coarsely sampling the cue-target onset asynchronies.^61^^,^^62^^,^^63^ At the same time, this value, corresponding to ≈17.7 cm/s in the SC, is in a similar range as the speed of proposed attentional scanning estimated in a recent opinion article.^40^ Worth mentioning is that the situation is entirely different in the Step task. In the Step task, attention is automatically captured by the target onset, and requires no effortful endogenous shift from the fixation point to the target. Physiologically, the saccade target onset may evoke a spatial peak in the neuronal map of space, and this spatial peak appears abruptly at the location corresponding to the target; it does not need to shift there from the fovea. Thereby, attention in the Step task jumps from the fovea to the target. The vigor and speed with which this attention jump, and the subsequent saccade happens depends on the eccentricity, size, and contrast of the target – which are the effects observed for the Step task and modified Step task. But these effects are related to the direct translation of those target characteristics into saccade initiation, not related to endogenous attention shift.

Alternatively, the SRT increase in the Delayed task might reflect a speed-accuracy tradeoff. One way to describe the speed-accuracy tradeoff is to use the classical Fitts’s Law.^64^ According to Fitts’s Law ($M=A+B\;\log_{2}\left(\frac{2S}{D}\right)$, the movement time (M) increases linearly with the logarithm of the ratio between movement distance (S) and target diameter (D). If we consider M to be the SRT, S to be the target eccentricity, and D to be the target size, Fitts’s Law, however, cannot explain the SRT values observed across conditions. Specifically, the fact that at the same target eccentricity, Scaled targets yielded similar or even longer SRT compared to the Equal targets is not consistent with Fitss’s Law. One might instead take D to be the size of the invisible target window, which was the same in all conditions. But then, it is difficult to explain the effect of the scaling observed in the Step task. In both cases, Fitts’s Law struggles to explain the variability of SRT in our data. Note that Wu et al.^65^ found that Fitts’s Law describes the speed-accuracy tradeoffs in a saccade sequence only when the time for the secondary, corrective saccades is included. When only the saccade latency for the primary saccade is considered as what we did in our analysis, Fitts’s law no longer holds. Furthermore, within the context of a diffusion model, the speed-accuracy tradeoff is typically modeled by adjusting the decision threshold.^66^ With other parameters staying unchanged, a higher threshold implies both a longer response time and a larger variance in the response time distribution. However, we do not find that SRT mode relates consistently to FWHM. For the respective example monkey, in the Step task, scaling eliminated the eccentricity-dependent SRT mode increase but not the FWHM increase (Figure 2); in the delayed task, adding a foveal flash eliminated the FWHM increase but not the SRT mode increase (Figure 5). All these observations indicate that a speed-accuracy tradeoff, at least in isolation, is unlikely to explain the observed SRT variation.

If the pre-saccadic attention shift caused the SRT increase in the Delayed task, one would expect to see a similar SRT increase for medium-sized saccades in the delayed visually guided saccades task of Hafed and Goffart.^4^ Indeed, although the SRT increase in their delayed task is less prominent than in their Step task, it is clearly trending. The relatively small SRT increase in the delayed task is also apparent in the present study (comparing Figures 5 and 6 to Figures 2 and 3). In addition, it is worth noting that our SRT values are generally longer than those reported by Hafed and Goffart.^4^ This discrepancy is likely due to the smaller target windows used in our study (1.0 dva vs. 2–2.5 dva in radius). The size of the fixation window may be related to the speed-accuracy tradeoff as discussed earlier. Although the process involved in the speed-accuracy tradeoff may not be the direct cause of the SRT increase, it is still worth exploring how these factors interact.

### Variability of saccadic reaction times

As noted in previous studies, SRT exhibited significant trial-by-trail variation in both the Step and Delayed tasks. Within the framework of a rise-to-threshold model, a larger variance in SRT means either a higher threshold or a more variable rate of rise.^13^^,^^23^^,^^24^^,^^25^ A consistent finding in our study is the increased variability of SRT for targets located in the periphery. This pattern held true for both Equal and Scaled targets in both the step and delayed tasks. Notably, in the Step task with Scaled targets, SRT variance increased with target eccentricity, despite the effective flattening of the SRT mode by scaling (Figures 2 and 3). These findings suggest that factors beyond foveal magnification, and to some degree invariant to the task, contributed to the observed SRT variance increase with target eccentricity. One potential mechanism could be that neuronal response fields (both motor and visual) of larger eccentricity typically have a larger size. Larger response field sizes likely lead to less specific, or in other words, noisier contributions to the ensuing saccade. When cortical magnification is controlled, this leads to higher noise in the recruited neuronal population. Interestingly, Hafed and Chen^67^ reported a weaker and more variable visual response associated with less accurate saccades in SC neurons with a larger response field. Note that these authors compared neurons representing lower versus upper visual fields, yet similar properties are conceivable by comparing neurons with response fields centered at different eccentricities.

### Individual differences

We observed considerable variations in SRT distributions among the four tested monkeys. These variations likely have multiple sources. First, some variance in SRT distributions could be related to the task design. Most prominently, the maximum SRT allowed was 500 ms at all locations in all tasks. This setting is essential for assuring the same task structure across all conditions despite the difference in their typical SRTs. However, this setting is not optimal for eliminating the influence of task-irrelevant processes, e.g., procrastinations to reduce the landing errors. Indeed, the monkeys whose SRTs showed the largest variance, suggesting extra noise in their decision process, were also the ones that seemed to be outliers (HU in Figure 2C and HO in Figure 4C). Since using a long response window is required to compare SRT distributions that are expected to shift significantly, future studies can use variable reward amounts to encourage the animal to make as fast a response as possible and potentially reduce the variability among the subjects.^68^ Second, variations associated with scaling might be partially explained by the individual differences in foveal magnification factors.^69^ Finally, it is conceivable that some of the monkeys, e.g., monkey HU, had comparatively poorer vision. Overall, the vision was sufficient for all tested monkeys to produce the observed, consistent behavior. However, subtle deficiencies in vision might have contributed to the observed individual differences, and the individual visual acuity was not tested here.

Increasing the number of subjects is crucial for obtaining a more comprehensive understanding of whether the observed effect is present in the population. However, in the current study, the number of subjects was limited, partly because of the chosen animal model, i.e., macaques. Macaque monkeys were used due to the availability of published data on the foveal magnification factor in the SC, which was essential for the experimental design. Additionally, there is a rich history of using macaque monkeys to study the underlying neural mechanisms of oculomotor control and attention. Choosing macaque monkeys thus offers the potential to use these findings to motivate future circuit investigations. In human studies, it is generally easier to include a larger number of subjects; however, there is no direct measurement of the SC foveal magnification in humans. Nevertheless, it is not unreasonable to utilize the same magnification factor as observed in macaque monkeys. Alternatively, one can consider using the magnification factor measured for the primary visual cortex, as evidence indicates that the magnification factor of the primary visual cortex and SC are similar.^17^

### Limitations of the study

Our ability to draw inferences about the population is limited by the small sample size. To increase the sample size, we have suggested conducting similar experiments with human participants. Using human participants would also allow us to adopt more sophisticated task designs to address other limitations of the study. For example, our explanation of the SRT-eccentricity relationship in the Step task centers on target visibility. However, SRT reflects the total time required for target detection, response initiation, and execution. A dissociation of these components, respectively, their dependence on eccentricity, might have been achieved by, e.g., including a condition with a manual response that remains identical across eccentricities.^4^ Such a dissociation is beyond the scope of the present study but is an interesting target for future research. In the Delayed task, our argument assumed the obligatory shift of attention prior to saccade execution.^39^^,^^51^ However, the time course of attentional deployment to either the target or intermediate locations was not tested explicitly. A dual-task design combining a detection or discrimination task along with the saccade task may provide more direct evidence of an attentional scan. Finally, we have limited the target positions to the right horizontal meridian. These positions share the same polar angle but differ in eccentricity, making them well-suited for investigating the role of foveal magnification. Similar to the magnified foveal representation, the representation of the upper visual field is also magnified in the SC.^67^ Therefore, it would be interesting to include target locations of varying polar angles to test whether the proposed mechanism generalizes to other forms of asymmetries in the SC map.

## Resource availability

### Lead contact

Requests for further information and resources should be directed to and will be fulfilled by the lead contact, Yufeng Zhang (yufeng.zhang42@outlook.com).

### Materials availability

This study did not generate new unique reagents.

### Data and code availability

• Raw data reported in this article will be shared by the lead contact upon request.
• All original code and preprocessed data necessary for replicating the results contained in this study have been deposited at Zenodo and are publicly available as of the date of publication. The relevant DOI is listed in the key resources table.
• Any additional information required to reanalyze the data reported in this article is available from the lead contact upon request.

## Acknowledgments

This work was supported by 10.13039/501100001659DFG (FOR 1847 FR2557/2-1, FR2557/5-1-CORNET, FR2557/7-1-DualStreams to P.F.), 10.13039/100006826EU (HEALTH-F2-2008-200728-BrainSynch, FP7-604102-HBP to P.F.), a European Young Investigator Award to P.F., 10.13039/100000002National Institutes of Health (1U54MH091657-WU-Minn-Consortium-HCP to P.F.), the LOEWE program (NeFF to P.F.).

The authors would like to thank Ziad Hafed for helpful discussions about the study, Tim Näher for advice on data analysis, Jackson Smith for training of monkey HO, and Sabrina Wallrath, Julia Hoffmann, and Marianne Hartmann for technical support with monkey training and behavioral data collection. The authors would also like to thank the reviewers for their valuable feedback during the revision process.

## Author contributions

YZ: Conceptualization, methodology, software, validation, formal analysis, investigation, data curation, writing – original draft, and writing-reviewing and editing; PF: Methodology, validation, resources, writing – original draft, writing-reviewing and editing, supervision, and funding acquisition.

## Declaration of interests

P.F. has a patent on thin-film electrodes and is a member of the Advisory Board of CorTec GmbH (Freiburg, Germany). The authors declare no further competing interests.

## Declaration of Generative AI and AI-assisted technologies in the writing process

During the preparation of this work, the authors used ChatGPT and DeepL in order to enhance the article’s grammatical correctness and overall readability. After using these tools, the authors reviewed and edited the content as needed and take full responsibility for the content of the publication.

## STAR★Methods

### Key resources table

**Table:** 

REAGENT or RESOURCE	SOURCE	IDENTIFIER
**Experimental models: Organisms/strains**		

Macaque monkeys (macaca mulatta)	Medical Research Council Centre for Macaques, Porton Down, Salisbury, SP4 0JQ	N/A

**Software and algorithms**		

ARCADE (Stimuli presentation and behavioral control)	GitHub	https://github.com/esi-neuroscience/ARCADE
MATLAB	MathWorks Inc.	MATLAB 2018b
Python	conda-forge	3.10.14
NumPy	conda-forge	1.26.4
SciPy	conda-forge	1.13.1
pandas	conda-forge	2.2.2
Matplotlib	conda-forge	3.8.4
statsmodels	conda-forge	0.14.2
patsy	conda-forge	0.5.6

**Deposited data**		

Processed data and analysis scripts	This study	https://doi.org/10.5281/zenodo.15686112

**Other**		

Eyelink 1000	SR Research Ltd.	https://www.sr-research.com/
Monitor	LG Corporation	Model 32GK850G-B

### Experimental model and study participant details

Four male adult monkeys (Macaca mulatta), aged 15–18 years, participated in the current study (referred to as CH, HO, KL and HU). All experimental procedures were conducted in compliance with the German and European animal protection laws. The experiments were approved by the responsible local authority, the Regierungspräsidium Darmstadt. All animals were implanted with a titanium headpost.^70^ Additionally, KL and HO had recording chambers implanted for addressing other scientific questions. The animals’ water intake was regulated to ensure their motivation during the behavioral task in which a small juice reward was provided after each correct trial. All animals were trained on the Step task. For this task, we found consistent effects, described in the Results section, for monkeys CH, HO and KL, but not for HU. We decided to investigate the effects found in CH, HO and KL in more detail in the Delayed task. As those effects had not been observed in HU, this monkey was excluded from the Delayed task. After data collection on the Delayed task, HO was additionally trained on the modified Step task.

### Method details

#### Visual stimuli

All stimuli were controlled by custom software (https://github.com/esi-neuroscience/ARCADE) and presented on an LCD monitor (LG 32GK850G-B) at 143.9Hz. Viewing distance was 78 cm for all monkeys. Precise stimulus presentation time was validated with a photodiode attached to the screen. Parameters for all stimuli used are listed below.

*Fiation dot.* Trials in all tasks started with presenting a fixation dot. The fixation dot in all trials was a white filled cycle of 0.1 dva diameter, 243.7cd/m^2^, at the center of the screen.

*Target stimuli.* Each trial contained a single target stimulus chosen randomly from a predetermined set. As explained in the introduction, we focused on medium-sized saccades between 2 and 10 dva. In addition, to increase the trial counts per condition, we limited the saccade targets to the right visual field. Specifically, the stimulus set in the Step and the Delayed task contained targets centered at 2, 3, 4, 5, 6, 8, and 10 dva along the horizontal median to the right of the screen. At each target location, the set contained one target belonging to the *Equal* group and one target belonging to the *Scaled* group. For the *Equal* group, the target was a white filled circle of 243.7 cd/m^2^ and 0.1 dva diameter at all eccentricities. For the *Scaled* group, the target was a white filled circle of 243.7 cd/m^2^ and 0.1, 0.13, 0.17, 0.20, 0.24, 0.31, 0.38 dva diameters at 2, 3, 4, 5, 6, 8, and 10 dva, respectively. The size of the scaled target was calculated based on the following mapping from visual coordinates to the SC tissue space reported in Chen et al.^17^:

$$X=1.1\cdot \log_{e}\left(\frac{\sqrt{R^{2}+1.8\cdot Rcos\left(\theta \right)+0.81}}{0.9}\right)$$

$$Y=1.8\cdot \arctan\left(\frac{Rsin\left(\theta \right)}{Rcos\left(\theta \right)+0.9}\right)$$

where X is the anatomical coordinate representing the horizontal median, Y is the orthogonal anatomical coordinate, R is the eccentricity in dva and θ is the angle in degrees from the horizontal median.

All target stimuli are small relative to the spacing between them. In the modified Step task with variable target contrast, the target locations were limited to 2, 4, and 6 dva, and at each eccentricity and for both *Scaled* and *Equal* targets, the possible target set contained targets of three different luminance levels: namely low contrast, 74.57 cd/m^2^; medium contrast, 124.66 cd/m^2^ and high contrast 243.7 cd/m^2^.

*Screen background.* Gray background was used in all tasks. Except for the modified Step task with variable target contrast, the screen background luminance was 60.0 cd/m^2^. In the modified Step task, the screen background luminance was 30.7 cd/m^2^.

*Foveal flash.* The foveal flash used in the Delayed task was a white filled circle of 1.0 dva and 243.7 cd/m^2^.

#### Behavioral tasks

We recorded the binocular gaze data at 500 Hz with an Eyelink-1000 system (SR Research Ltd.). Real-time gaze data from one eye was used for online behavioral control. The real-time monitoring of response saccade initiation used a virtual fixation window (r ≈ 0.8 dva) centered on the fixation dot. Saccade landing on the target was determined by gaze entering and staying in a virtual target window (r ≈ 1.0 dva) centered on the saccade target. The target window radius was the same in all conditions. Additionally, the saccade flight time between leaving the fixation window and entering the target window was monitored and limited to less than ≈100 ms. In all tasks, trials of different conditions were randomly interleaved.

In the Step task (Figure 1B), after fixation was acquired and maintained for 800 to 1500 ms, the fixation dot was removed, and in the same video frame, a single target was presented in the periphery. The animal needed to make a saccade, within 500 ms, to the target and hold its gaze on the target for another 800 to 1500 ms to obtain the juice reward.

In the Delayed task (Figure 1C), after the fixation was acquired and maintained for 800 to 1000 ms, without removal of the fixation point, a single target was presented in the periphery for 800 to 1500 ms after which the fixation dot was removed. Only after fixation dot removal, the monkey was allowed to make a saccade to the target, and it needed to make a saccade towards the target within 500 ms and hold its gaze on the target for another 800 to 1500 ms to successfully complete the trial and obtain the reward. Additionally, in 50% of the trials in the Delayed task, we included a ≈30 ms flash at the fovea (white-filled circle of 1.0 dva diameter) presented ≈100 ms after the fixation dot offset. The flash had no task relevance, and the monkey needed to complete the trial as in trials without flash.

#### Trial inclusion

Only correct trials were included. As described above, in these trials, the monkey made timely (SRT<500 ms) and relatively precise (error below ≈1.0 dva) primary saccades to the target. Additionally, 1) Trials with SRTs less than 100 ms were excluded from all tasks. 2) In Delayed task trials containing a foveal flash, any trial with the response saccade occurring within 100 ms after the flash was also excluded.

#### Saccade detection and SRT definition

Saccades were detected using a two-dimensional velocity threshold, following the methodology described in Engbert and Kliegl.^71^ In our analysis, we set lambda to 10. Saccade onset time was determined as the moment when the velocity threshold was crossed. In all tasks, SRT was defined as the time elapsed between the offset of the fixation dot and the onset of the response saccade.

### Quantification and statistical analysis

The regression analysis was performed with the Python module statsmodels.^72^ Further details can be found in the corresponding Results section. We used a permutation test, permuting the respective regressor label (*N* = 1000), to test against the null hypothesis that the coefficient equals zero. The two-tailed *p*-values calculated from the resulting permutation distribution are reported in the main text. Bootstrapping (*N* = 1000) was used to calculate the confidence interval. Each bootstrap sample was obtained by resampling trials within each group of trials corresponding to individual data points in the respective regression analysis.

### Additional resources

This study is not part of a clinical trial.
