# Supplementary material for: Eccentricity-dependent saccadic reaction time: The roles of foveal magnification and attentional orienting
Source: iScience. 2025 Jul 1;28(8):113042. doi: 10.1016/j.isci.2025.113042 (PMC12281140; doi:10.1016/j.isci.2025.113042)
Supplement: Document S1. Figures S1–S8 [file mmc1.pdf]

iScience, Volume *28*

Supplemental information

Eccentricity-dependent saccadic reaction time: The roles of foveal magnification and attentional orienting

Yufeng Zhang (张宇峰) and Pascal Fries

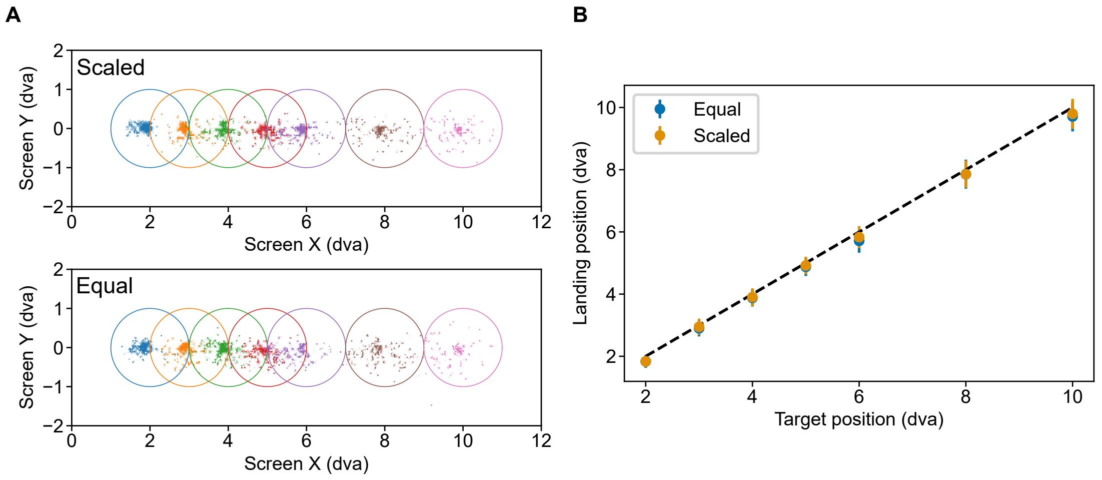

Figure S1. Saccade landing positions for monkey HO in the Step task, related to Figure 2. (A) Scatter plot for the primary saccade landing positions. The dotted circles illustrate the virtual target window (r ≈ 1.0 dva). Note that a small number of landing positions appear outside the dotted circles. This is most likely due to differences between the offline data analysis shown here and the online experimental control illustrated by the dotted circles. (B) Average landing positions plotted against the actual target position. Error bars represent the standard deviation.

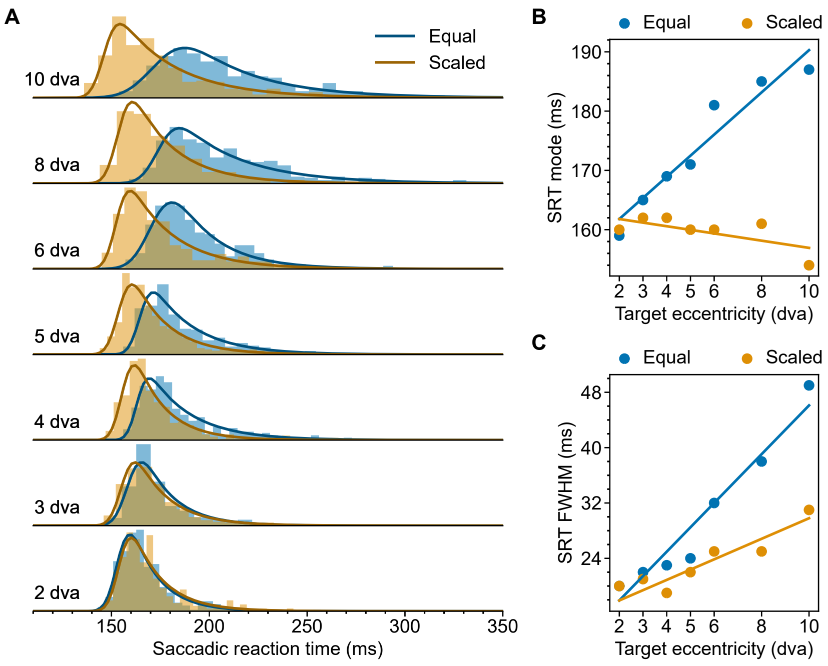

Figure S2. Eccentricity-dependent SRT in the Step task in Monkey CH, related to Figure 2. **(A)** SRT distributions for Equal (Blue) and Scaled (Orange) targets. Solid lines represent the fitted ex-Gaussian distributions. **(B)** Multiple linear regression of SRT mode on target eccentricity and scaling ($Equal, CI_{\beta_1,95\%} = [2.99,\ 4.20], p < .001$; $Scaled, CI_{\beta_1,95\%} = [-0.98, -0.34],\ p < .001$; $Combined,\ CI_{\beta_2,95\%} = [-4.67,\ -3.72],\ p < .001$). **(C)** Same as (B) but for FWHM ($Equal, CI_{\beta_1,95\%} = [3.05,\ 4.46], p < .001$; $Scaled, CI_{\beta_1,95\%} = [0.91,\ 1.79],\ p < .001$; $Combined,\ CI_{\beta_2,95\%} = [-2.52,\ -1.38],\ p < .001$).

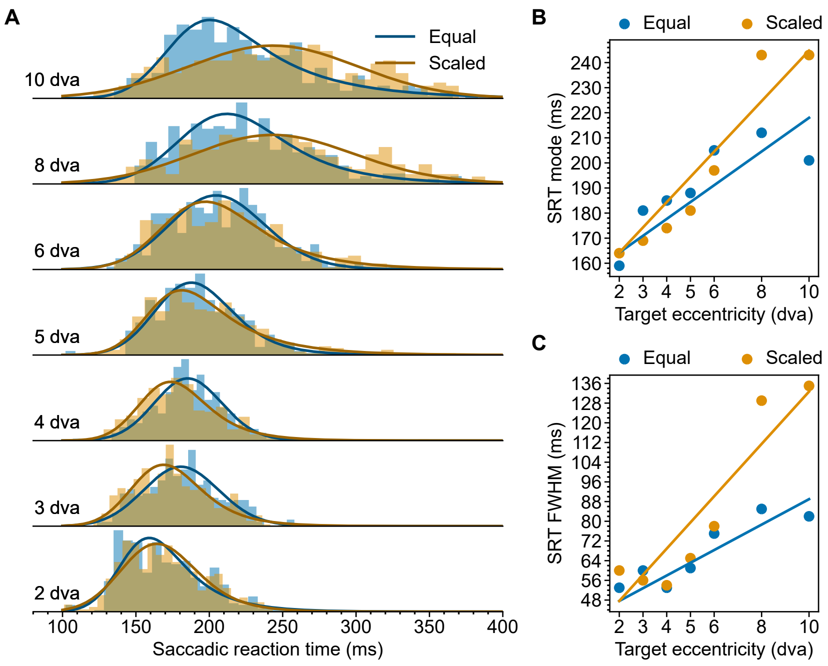

Figure S3. Eccentricity-dependent SRT in the Step task in Monkey HU, related to Figure 2. **(A)** SRT distributions for Equal (Blue) and Scaled (Orange) targets. Solid lines represent the fitted ex-Gaussian distributions. **(B)** Multiple linear regression of SRT mode on target eccentricity and scaling ($Equal, CI_{\beta_1,95\%} = [4.06,\ 6.56], p <..001$; $Scaled, CI_{\beta_1,95\%} = [10.30,\ 12.52],\ p < .001$; $Combined,\ CI_{\beta_2,95\%} = [2.17,\ 4.45],\ p < .001$). **(C)** Same as (B) but for FWHM ($Equal, CI_{\beta_1,95\%} = [3.13,\ 5.88], p < .001$; $Scaled, CI_{\beta_1,95\%} = [9.92,\ 12.62]$, $p < .001$; $Combined,\ CI_{\beta_2,95\%} = [4.05,\ 6.55],\ p < .001$).

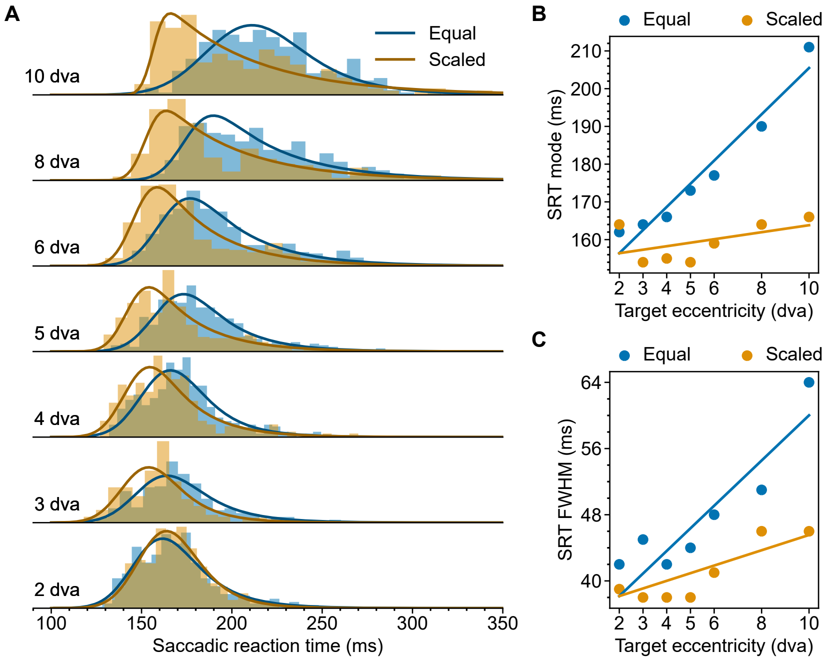

Figure S4. Eccentricity-dependent SRT in the Step task in Monkey KL, related to Figure 2. **(A)** SRT distributions for Equal (Blue) and Scaled (Orange) targets. Solid lines represent the fitted ex-Gaussian distributions. **(B)** Multiple linear regression of SRT mode on target eccentricity and scaling ($Equal, CI_{\beta_1,95\%} = [5.04,\ 7.18], p < .001$; $Scaled, CI_{\beta_1,95\%} = [0.51,\ 1.45],\ p < .001$; $Combined,\ CI_{\beta_2,95\%} = [-6.16,\ -4.48],\ p <$ $..001$). **(C)** Same as (B) but for FWHM ($Equal, CI_{\beta_1,95\%} = [1.52,\ 3.69], p <..001$; $Scaled, CI_{\beta_1,95\%} = [0.41,\ 1.80]$, $p < .001$; $Combined,\ CI_{\beta_2,95\%} = [-2.94,\ -1.09],\ p = .007$).

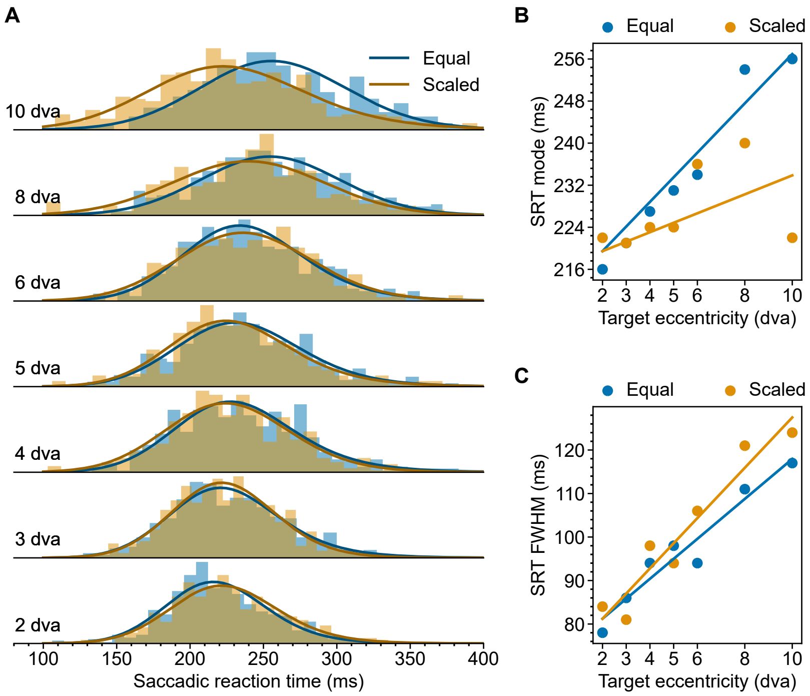

Figure S5. Eccentricity-dependent SRT in the Delayed task without foveal flash in Monkey CH, related to Figure 4. **(A)** SRT distributions for Equal (Blue) and Scaled (Orange) targets. **(B)** Multiple linear regression of SRT mode on target eccentricity and scaling ($Equal, CI_{\beta_1,95\%} = [4.24,\ 6.20], p < .001$; $Scaled, CI_{\beta_1,95\%} = [.12,\ 2.06],\ p = .011$; $Combined,\ CI_{\beta_2,95\%} = [-3.71,\ -1.83],\ p < .001$). **(C)** Same as (B) but for FWHM ($Equal, CI_{\beta_1,95\%} = [3.06,\ 5.67], p < .001$; $Scaled, CI_{\beta_1,95\%} = [4.30,\ 7.03],\ p < .001$; $Combined,\ CI_{\beta_2,95\%} = [.06,\ 2.59],\ p = .053$).

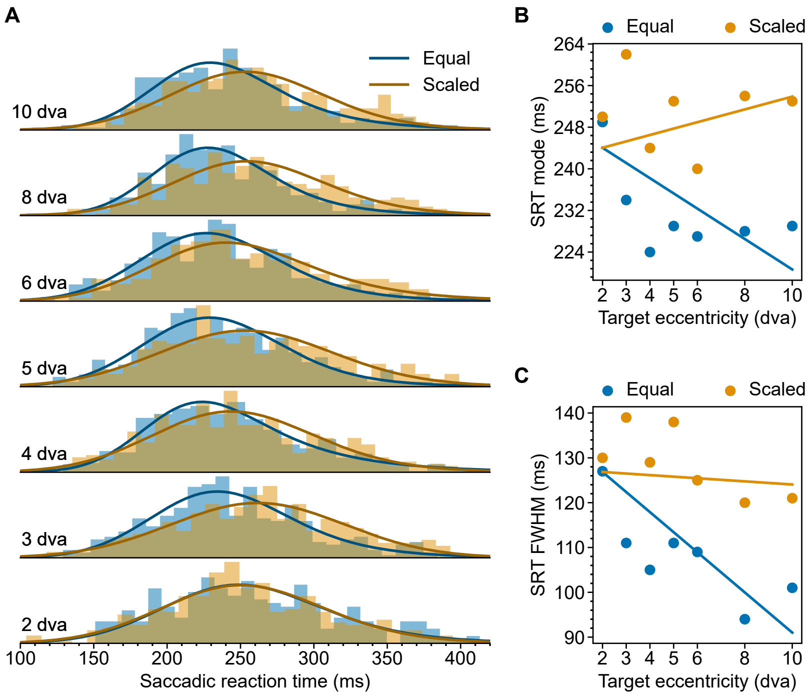

Figure S6. Eccentricity-dependent SRT in the Delayed task without foveal flash in Monkey HO, related to Figure 4. **(A)** SRT distributions for Equal (Blue) and Scaled (Orange) targets. **(B)** Multiple linear regression of SRT mode on target eccentricity and scaling ($Equal, CI_{\beta_1,95\%} = [-3.11, -.08], p = .018$; $Scaled, CI_{\beta_1,95\%} = [-1.77,\ 1.41],\ p = .935$; $Combined,\ CI_{\beta_2,95\%} = [2.75,\ 5.19],\ p < .001$). **(C)** Same as (B) but for FWHM ($Equal, CI_{\beta_1,95\%} = [-4.73,\ -1.03], p = .001$; $Scaled, CI_{\beta_1,95\%} = [-4.09,\ -.13],\ p = .061$; $Combined,\ CI_{\beta_2,95\%} = [2.33,\ 5.47],\ p < .001$).

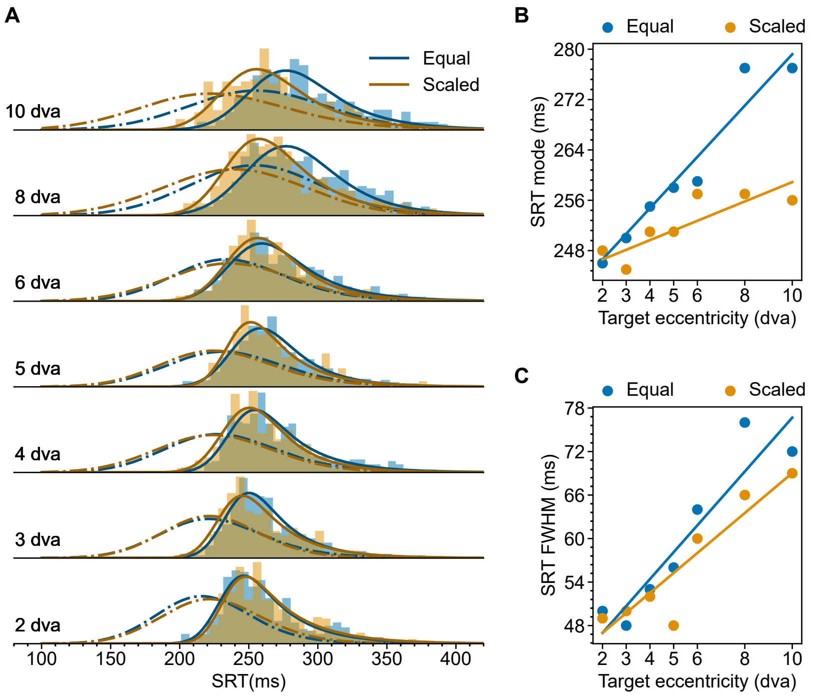

Figure S7. Eccentricity-dependent SRT in the Delayed task with foveal flash in Monkey CH, related to Figure 5. **(A)** SRT distributions for Equal (Blue) and Scaled (Orange) targets. Dashed lines represent the ex-Gaussian fits for SRT distributions in corresponding conditions without the foveal flash (as in Figure S5). **(B)** Multiple linear regression of SRT mode on target eccentricity and scaling ($Equal, CI_{\beta_1,95\%} = [3.31,\ 5.12], p <$ $.001$; $Scaled, CI_{\beta_1,95\%} = [.52,\ 2.29],\ p < .001$; $Combined,\ CI_{\beta_2,95\%} = [-3.41, -1.81],\ p < .001$). **(C)** Same as (B) but for FWHM ($Equal, CI_{\beta_1,95\%} = [2.62,\ 4.85],\ p < .001$; $Scaled, CI_{\beta_1,95\%} = [1.82,\ 3.96],\ p <$ $.001$; $Combined,\ CI_{\beta_2,95\%} = [-1.96,\ .12],\ p = .075$).

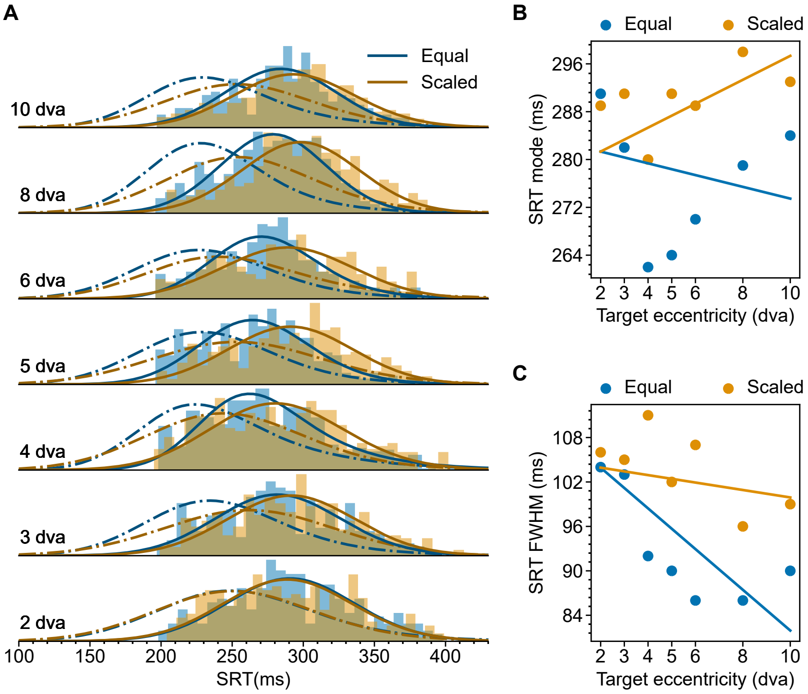

Figure S8. Eccentricity-dependent SRT in the Delayed task with foveal flash in Monkey HO, related to Figure 5. **(A)** SRT distributions for Equal (Blue) and Scaled (Orange) targets. Dashed lines represent the ex-Gaussian fits for SRT distributions in corresponding conditions without the foveal flash (as in Figure S6). **(B)** Multiple linear regression of SRT mode on target eccentricity and scaling ($Equal, CI_{\beta_1,95\%} = [-.68,\ .93], p = .999$; $Scaled, CI_{\beta_1,95\%} = [.201,\ 2.26],\ p = .012$; $Combined,\ CI_{\beta_2,95\%} = [2.38,\ 3.65],\ p < .001$). **(C)** Same as (B) but for FWHM ($Equal, CI_{\beta_1,95\%} = [-2.97,\ -0.69],\ p = .001$; $Scaled, CI_{\beta_1,95\%} = [-2.29,\ .05],\ p = .021$; $Combined,\ CI_{\beta_2,95\%} = [1.35,\ 3.00],\ p < .001$).
